# Using structural equation modeling to investigate change and response shift in patient-reported outcomes: practical considerations and recommendations

**DOI:** 10.1007/s11136-020-02742-9

**Published:** 2021-02-07

**Authors:** M. G. E. Verdam, F. J. Oort, M. A. G. Sprangers

**Affiliations:** 1 Department of Methodology and Statistics, Institute of Psychology, Leiden University, P.O. Box 9555, 2300 RB Leiden, The Netherlands; 2 Department of Medical Psychology, Amsterdam University Medical Centre, Amsterdam Public Health Research Institute, Amsterdam, The Netherlands; 3 Research Institute Child Development and Education, University of Amsterdam, Amsterdam, The Netherlands

**Keywords:** Structural equation modeling (SEM), Patient-reported outcomes (PROs), Health-related quality of life (HRQL), Change, Response shift

## Abstract

**Background:**

Patient-reported outcomes (PROs) are of increasing importance for health-care evaluations. However, the interpretation of change in PROs may be obfuscated due to changes in the meaning of the self-evaluation, i.e., response shift. Structural equation modeling (SEM) is the most widely used statistical approach for the investigation of response shift. Yet, non-technical descriptions of SEM for response shift investigation are lacking. Moreover, application of SEM is not straightforward and requires sequential decision-making practices that have not received much attention in the literature.

**Aims:**

To stimulate appropriate applications and interpretations of SEM for the investigation of response shift, the current paper aims to (1) provide an accessible description of the SEM operationalizations of change that are relevant for response shift investigation; (2) discuss practical considerations in applying SEM; and (3) provide guidelines and recommendations for researchers who want to use SEM for the investigation and interpretation of change and response shift in PROs.

**Conclusion:**

Appropriate applications and interpretations of SEM for the detection of response shift will help to improve our understanding of response shift phenomena and thus change in PROs. Better understanding of patients’ perceived health trajectories will ultimately help to adopt more effective treatments and thus enhance patients’ wellbeing.

**Supplementary Information:**

The online version contains supplementary material available at 10.1007/s11136-020-02742-9.

## Introduction

Patient-reported outcomes (PROs) are increasingly recognized as a critical endpoint in health care and medicine, and routine assessment of PROs is becoming standard part of clinical practice [1]. The importance of measuring PROs, such as health-related quality of life (HRQL), is especially salient in view of aging societies and more powerful health-care interventions, which have led to an increasing number of people living with chronic disease [2]. That is, the ultimate purpose of health-care interventions may often not be prolonged survival but maintenance or optimization of patients’ quality of life [3].

Evaluating the impact of disease and treatment on patients’ perceived health trajectories requires longitudinal assessment. However, interpretation of change in PROs is complicated by the fact that the meaning of respondents’ self-evaluations may change too. Sprangers and Schwartz [4] proposed a theoretical model for change in the meaning of self-evaluations, which they called ‘response shift,’ a term coined by Howard et al. [5]. Sprangers and Schwartz distinguish three types of response shift: *recalibration* refers to a change in respondents’ internal criteria with which they assess the construct of interest; *reprioritization* refers to a change in respondents’ values regarding the relative importance of subdomains; and *reconceptualization* refers to a change in the meaning of the target construct. Although various refined definitions and theoretical models have subsequently been proposed [6–8], they all share this working definition of response shift. While response shift can often be considered a beneficial treatment or time effect, its effect may lead to an over- or under-estimation of intervention effects, hindering the interpretation of change in HRQL outcomes. It is, thus, important to detect and take into account possible response shift effects.

Structural equation modeling (SEM) is currently the most widely used statistical approach for the investigation of response shift [9] and has been applied to examine response shift in various patient populations, disease types, and PRO measures. However, application of SEM is relatively complex as it includes many steps that require several decisions regarding, for example, the number and types of response shift to consider. When one is not aware of these different decisions and their consequences, there is a risk of using the SEM method inappropriately. Moreover, there is a lack of non-technical explanations of SEM for response shift detection; the original paper by Oort [10] is difficult to follow for non-statisticians as it contains many technical specifications, makes an unnecessary distinction between two types of recalibration, and distinguishes several other types of change that are not directly relevant for response shift investigation. The aim of the current paper is therefore to provide an accessible description of the SEM method and its associated sequential decision-making practices, in order to stimulate valid applications and interpretations of SEM for the investigation of response shift and change in HRQL outcomes. Specifically, we describe the operationalization and interpretation of change with SEM addressing only those parameters of interest for the detection of recalibration, reprioritization, and reconceptualization response shift and ‘true’ change in the target construct (i.e., change in the target construct while taking into account response shift), and discuss practical considerations in the application of the SEM approach. In doing so, we provide guidelines and recommendations for the investigation and interpretation of change and response shift.

Our paper is targeted at researchers who are interested in applying SEM for response shift detection and are familiar with latent variable modeling (e.g., see [11]). Note that SEM can be used to investigate response shift from both a conceptual and a measurement perspective (see [9] for formal definitions of both perspectives). In the current paper, we address response shift investigation from the measurement perspective, where response shift is defined as a change in the relation between the underlying (latent) target construct (e.g., HRQL) and the observed questionnaire responses. To explain the SEM method, enhance its accessibility, and facilitate the interpretation of its results, we use an example of HRQL measurement over time. However, we would like to emphasize that response shift can occur—and be investigated—in any PRO measure (PROM).

## Operationalization and interpretation of change and response shift

**Fig. 1:**
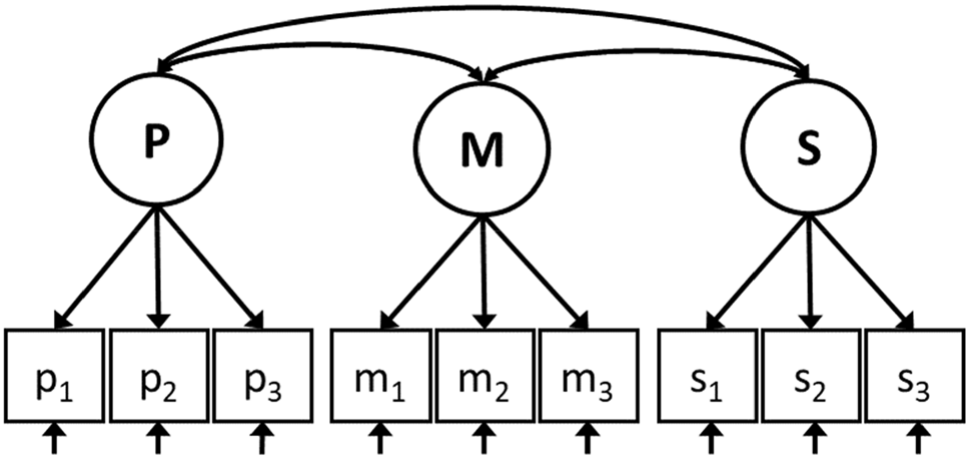
A SEM model for physical (P), mental (M), and social (S) health. The squares at the bottom represent nine observed indicators, where *p*_1_ to *p*_3_ refer to the three measures of physical health (i.e., ‘nausea,’ ‘pain,’ and ‘fatigue’), *m*_1_ to *m*_3_ refer to the three measures of mental health (i.e., ‘anxiety,’ ‘sadness,’ and ‘happiness’) and *s*_1_ to *s*_3_ refer to the three measures of social health (i.e., ‘family relations,’ ‘friendships,’ and ‘work relations’). The solid single-headed arrows at the bottom of the squares represent the residual factors of each indicator variable. The circles at the top represent the underlying latent variables that measure everything that the indicators that load on that factor have in common [i.e., a physical (P), mental (M), and social (S) domain of HRQL]. Each arrow from a latent variable to an observed indicator represents a factor loading. The solid double-headed arrows between the latent variables represent common factor covariances

Suppose cancer patients are administered a HRQL questionnaire prior to and at the end of chemotherapy. We have their scores on nine different items from a HRQL questionnaire that measures physical (i.e., ‘nausea,’ ‘pain,’ and ‘fatigue’), mental (‘anxiety,’ ‘sadness,’ and ‘happiness’) and social (‘family relations,’ ‘friendships,’ and ‘work relations’) aspects of health. SEM is a statistical technique that can be used to model relationships between observed responses (e.g., patients’ scores on the nine items of the HRQL questionnaire) to be reflective of one or more unobserved latent variables or common factors (e.g., the three domains of the HRQL construct that the items aim to measure) (see Fig. 1). Within the SEM framework, the variances and covariances (**Σ**, ‘Sigma’) and means (**μ**, ‘mu’) of the observed variables (*X*) are given by

$${\text{Cov}}\left( {X,X^{\prime}} \right) = {{\varvec{\Sigma}}} = {{\varvec{\Lambda}}} \, {{\varvec{\Phi}}} \, {\varvec{\Lambda^{\prime}}} + {{\varvec{\Theta}}},$$

and

$${\text{Mean}}\left( X \right) = {{\varvec{\upmu}}} = {{\varvec{\uptau}}} + {{\varvec{\Lambda}}} \, {{\varvec{\upkappa}}},$$

where **Λ** (‘Lambda’) is a matrix of common factor loadings that describes the relationships between the observed variables and underlying common factors (e.g., the relationships between the underlying common factor mental health and the three associated item scores are specified by three common factor loadings), **Φ** (‘Phi’) is a matrix of common factor variances and covariances that describes the relationships between the underlying factors (e.g., the relations between physical, mental, and social health), **Θ** (‘Theta’) is a matrix of residual variances and covariances that cannot be explained by the underlying common factors (e.g., the variances of the nine observed item scores that cannot be explained by the three underlying common factors), **τ** (‘tau’) is a vector of intercepts (e.g., one intercept value for each of the nine item scores), and **κ** (‘kappa’) is a vector of common factor means (e.g., the means of the underlying common factors physical, mental, and social health). The full matrices of the SEM model for the example from Fig. 1 are provided in Online Appendix A.

### Assessment of different types of change

**Fig. 2:**
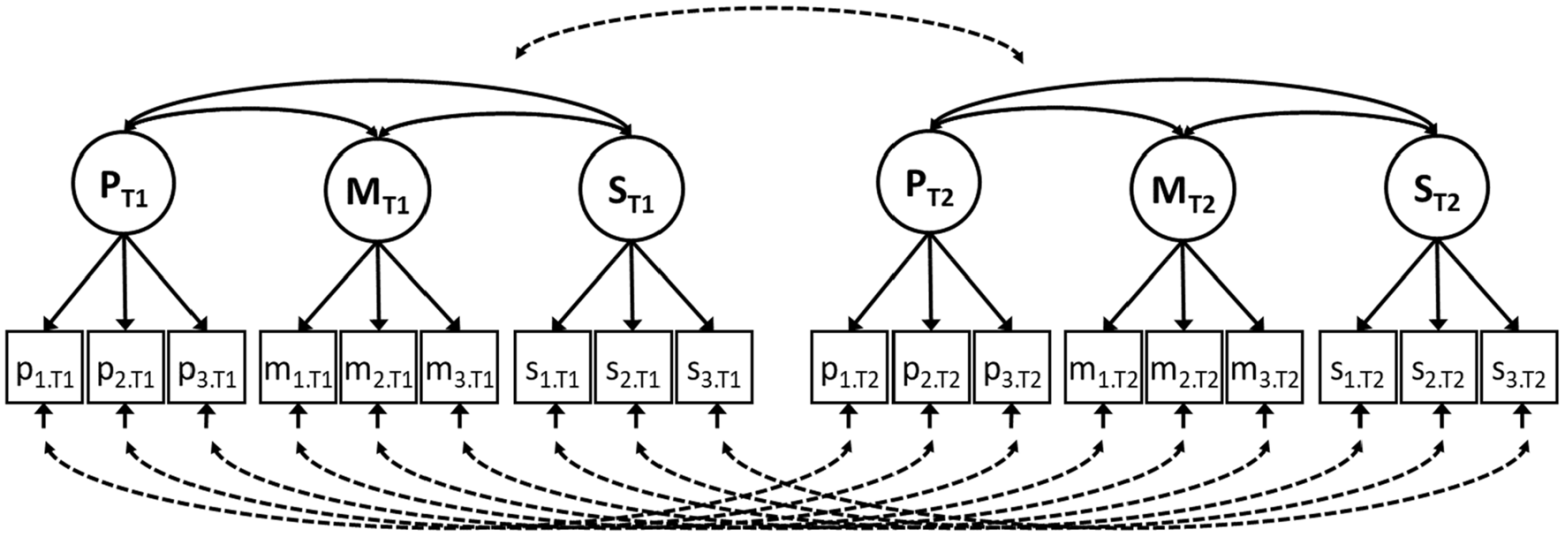
A longitudinal SEM model for the investigation of change and response shift in physical (P), mental (M), and social (S) health. This is the longitudinal SEM model of the same HRQL measurement as depicted in Fig. 1. The squares at the bottom represent the observed indicators, measuring physical (*p*_1_ to *p*_3_), mental (*m*_1_ to *m*_3_), and social (*s*_1_ to *s*_3_) aspects of health (see Fig. 1) at two occasions (T1 and T2). The solid single-headed arrows at the bottom of the squares represent the residual factors of each indicator variable. The dotted double-headed arrows represent the longitudinal relations between the residual factors, where only the residual factors of the same indicator are allowed to correlate. The circles at the top represent the underlying latent variables that measure everything that the indicators that load on that factor have in common [i.e., a physical (P), mental (M), and social (S) domains of HRQL, both at T1 and T2]. Each arrow from a latent variable to an observed indicator represents a factor loading. The solid double-headed arrows between the latent variables represent common factor covariances. The dotted double-headed arrow represents the (nine) longitudinal correlations between the common factors

SEM can be applied to data from multiple measurement occasions to assess change (see Fig. 2). Specifically, the SEM method for the investigation of different types of changes in HRQL outcomes [10] uses change in the pattern of factor loadings, values of factor loadings, and intercepts to operationalize reconceptualization, reprioritization, and recalibration, respectively. In the presence of response shift, the meaning of the construct is not consistent across time. In other words, a comparison of the indicators for which response shift has been detected is compromised, as change in the observed indicators does not (only) reflect change in the underlying variables. A decomposition of change can be used to investigate the impact of response shift on change in the observed indicators [12]. Moreover, SEM enables the investigation of change in the underlying latent variables, while taking into account possible response shifts. Changes in the common factor means across occasions are indicative of ‘true’ change in the construct of interest. Table 1 provides an overview of the four steps of the SEM approach as proposed by Oort [10], including examples of the interpretation of response shift.

**Table 1:** Illustration of response shift detection using the four-step structural equation modeling (SEM) procedure

The SEM approach	General example	Illustrative example
Step 1: establishing a measurement model		
$${\text{Cov}}\left( {X,X^{\prime}} \right) ={{\varvec{\Sigma}}}= {{\varvec{\Lambda}}}{{\varvec{\Phi}}}{\varvec{\Lambda^{\prime}}}+{{\varvec{\Theta}}},$$ $$\begin{aligned} {\text{where}} = {{\varvec{\Sigma}}} = & \left[ {\begin{array}{*{20}c} {{{\varvec{\Sigma}}}_{{{\bf{T}}1}} } & {{{\varvec{\Sigma}}}_{{{\bf{T}}1,{\bf{T}}2}} } \\ {{{\varvec{\Sigma}}}_{{{\bf{T}}2,{\bf{T}}1}} } & {{{\varvec{\Sigma}}}_{{{\bf{T}}2}} } \\ \end{array} } \right] \\ = & \left[ {\begin{array}{*{20}c} {{{\varvec{\Lambda}}}_{{{\bf{T}}1}} } & 0 \\ 0 & {{{\varvec{\Lambda}}}_{{{\bf{T}}2}} } \\ \end{array} } \right]\left[ {\begin{array}{*{20}c} {{{\varvec{\Phi}}}_{{{\bf{T}}1}} } & {{{\varvec{\Phi}}}_{{{\bf{T}}1,{\bf{T}}2}} } \\ {{{\varvec{\Phi}}}_{{{\bf{T}}2,{\bf{T}}1}} } & {{{\varvec{\Phi}}}_{{{\bf{T}}2}} } \\ \end{array} } \right]\left[ {\begin{array}{*{20}c} {{{\varvec{\Lambda}}}_{{{\bf{T}}1}} } & 0 \\ 0 & {{{\varvec{\Lambda}}}_{{{\bf{T}}2}} } \\ \end{array} } \right]^{^{\prime}} + \user2{ }\left[ {\begin{array}{*{20}c} {{{\varvec{\Theta}}}_{{{\bf{T}}1}} } & {{{\varvec{\Theta}}}_{{{\bf{T}}1,{\bf{T}}2}} } \\ {{{\varvec{\Theta}}}_{{{\bf{T}}2,{\bf{T}}1}} } & {{{\varvec{\Theta}}}_{{{\bf{T}}2}} } \\ \end{array} } \right] \\ \end{aligned}$$$${\text{Mean}}\left( X \right)\, = \,{{\varvec{\upmu}}}\, = \,{{\varvec{\uptau}}}\, + \,{{\varvec{\Lambda}}} \, {{\varvec{\upkappa}}},$$ $${\text{where}} = {{\varvec{\upmu}}} = \left[ {\begin{array}{*{20}c} {{{\varvec{\upmu}}}_{{{\bf{T}}{1}}} } \\ {{{\varvec{\upmu}}}_{{{\bf{T}}2}} } \\ \end{array} } \right] = \left[ {\begin{array}{*{20}c} {{{\varvec{\uptau}}}_{{{\bf{T}}1}} } \\ {{{\varvec{\uptau}}}_{{{\bf{T}}2}} } \\ \end{array} } \right] + \left[ {\begin{array}{*{20}c} {{{\varvec{\Lambda}}}_{{{\bf{T}}{1}}} } & 0 \\ 0 & {{{\varvec{\Lambda}}}_{{{\bf{T}}2}} } \\ \end{array} } \right]\left[ {\begin{array}{*{20}c} {{{\varvec{\upkappa}}}_{{{\bf{T}}1}} } \\ {{{\varvec{\upkappa}}}_{{{\bf{T}}2}} } \\ \end{array} } \right]$$	The factor model is used to model the relationships between the observed variables and one or more underlying latent variables, where the underlying latent variables represent everything that the observed measures have in common (e.g., perceived health or HRQL). With longitudinal assessment, e.g., baseline (T1) and follow-up (T2), the same factor model can be applied at each measurement occasion to arrive at a longitudinal measurement model	Suppose we have patients’ scores on nine observed indicators that measure physical, mental, and social aspects of health. We use a three-factor model to represent the measurement structure of the data (see Fig. 1). The same factor model can be applied at both baseline (T1) and follow-up (T2) to arrive at a longitudinal measurement model (see Fig. 2). The specification of the full SEM matrices are provided in Online Appendix B. Example syntaxes for application of the SEM approach using the software programs Lavaan [33] and LISREL [29] are provided as supplementary material
Step 2: overall test of response shift^a^		
**Σ** and ${\varvec{\upmu}}$ as in Step 1, but with: ${{\varvec{\Lambda}}}_{\bf{T}1}$ = ${{\varvec{\Lambda}}}_{\bf{T}2}$**,** and = ${{\varvec{\uptau}}}_{\bf{T}1}{{\varvec{\uptau}}}_{\bf{T}2}$	To test for the presence of response shift, all model parameters associated with response shift (i.e., factor loadings and intercepts) are restricted to be equal across occasions, e.g., across baseline and follow-up. When these restrictions are not tenable, we continue to investigate which variable is affected by which type of response shift in Step 3	In the illustrative example from Fig. 2, the test for the presence of response shift entails equality restrictions on all nine factor loadings and all nine intercepts across occasions
Step 3: response shift detection^a^		
Pattern $({{\varvec{\Lambda}}}_{\bf{T}1})$** ≠ **Pattern ${({\varvec{\Lambda}}}_{\bf{T}2})$	*Reconceptualization* When the common factor loading of an observed indicator becomes zero or significantly different from zero at follow-up, this means that this indicator is no longer part of the measurement of the underlying latent variable or becomes part of the measurement, respectively, indicating a shift in conceptualization	Suppose that the common factor loading of the observed indicator ‘work relations’ on physical health is significantly different from zero at follow-up. This indicates that the scores on the subscale ‘work relations’ become (at least partly) indicative of physical health. It may be, for example, that ‘work relations’ is interpreted as related to physical functioning at follow-up (but not at baseline)
${{\varvec{\Lambda}}}_{{{\bf{T}}1}} \ne {{\varvec{\Lambda}}}_{{{\bf{T}}2}}$	*Reprioritization* When the value of a common factor loading is smaller or larger at follow-up as compared to baseline, this indicates a shift in the meaning (less or more important) of the indicator to the measurement of the underlying factor	Suppose the common factor loading of the observed indicator ‘anxiety’ is larger at follow-up as compared to baseline. This indicates that patients’ scores on ‘anxiety’ become more important to the measure of mental health. It may be, for example that anxiety due to uncertainty about the effectiveness of treatment and/or the course of the disease play an increasingly important role for patients’ mental health
${{\varvec{\uptau}}}_{{{\bf{T}}1}} \ne {{\varvec{\uptau}}}_{{{\bf{T}}2}}$	*Recalibration* When the intercept value of an observed indicator changes over time, this indicates a shift in the meaning of the response categories (internal standards) of the indicator, where the same ‘true’ state on the underlying factor corresponds to lower or higher scores on the indicator	Suppose the intercept value of the observed indicator ‘nausea’ is lower at follow-up as compared to baseline. This indicates that the same physical health leads to lower scores on nausea at follow-up as compared to baseline. It may be, for example, that patients get used to the experience of nausea, and therefore rate the same health-experience lower at follow-up as compared to baseline
Step 4: true change assessment		
**Σ** and ${\varvec{\upmu}}$ as in Step 3, including possible response shift, and assess whether ≠ ${{\varvec{\upkappa}}}_{\bf{T}1}{{\varvec{\upkappa}}}_{\bf{T}2}$	*‘True’ change* When the common factor means are lower or higher at follow-up, this indicates that patients’ HRQL decreases or increases	*Suppose the common factor mean of physical health is lower at follow-up as compared to baseline. This indicates that patients’ physical health deteriorates over time*

**Σ** is a symmetric matrix that contains the variances and covariances of the observed variables (*X*); **Λ** is a matrix of common factor loadings, where **Λ**_T1_ and **Λ**_T2_ contain the factor loadings of baseline (T1) and follow-up (T2), respectively; **Φ** is a symmetric matrix of common factor (co)variances, where **Φ**_T1_ contains the common factor (co)variances at baseline, **Φ**_T2_ contains the common factor (co)variances at follow-up, **Φ**_T1,T2_ contains the common factor covariances across occasion, and **Φ**_T1,T2_ = **Φ**_T2,T1_; **Θ** is a matrix of residual (co)variances, where **Θ**_T1_ is a diagonal matrix with residual variances at baseline, **Θ**_T2_ is a diagonal matrix with residual variances at follow-up, **Θ**_T1,T2_ is a diagonal matrix with residual covariances across occasion, and **Θ**_T1,T2_ = ***Θ***_T2,T1_; **μ** is a vector that contains the means of the observed variables; **τ** is a vector of intercept values, where **τ**_T1_ and **τ**_T2_ contain the intercepts at baseline and follow-up, respectively; **κ** is a vector of common factor means, where **κ**_T1_ and **κ**_T2_ contain the means of the common factors at baseline and follow-up, respectively. In Step 1, the following restrictions apply for identification purposes: diag(**Φ**_T1_) = **I**, diag(**Φ**_T2_) = **I**, **κ**_T1_ = 0, and **κ**_T2_ = 0. In Step 2, identification restrictions diag(**Φ**_T1_) =* I*, and **κ**_T1_ = 0 are sufficient, so that diag(**Φ**_T2_) and **κ**_T2_ are free to be estimated. ^a^Although non-invariance of residual variances can be considered as a type of non-uniform recalibration (see [10]), the detection of non-uniform recalibration is not important for the investigation of mean change in the common factors and therefore not considered here

### Added value of the SEM approach

There are three main advantages of the SEM approach to investigate change in HRQL outcomes. First, it allows for an operationalization of different types of response shift. Second, it can account for the different types of response shift. Third, the flexibility of the SEM framework enables the inclusion of multiple measurements (e.g., analyze more extensive follow-up designs; see [13]), multiple groups (e.g., compare different patient groups based on disease, treatment, or patient characteristics; see [14]), multidimensional scales (e.g., include multiple HRQL domains, or other latent variables, simultaneously; see [15]), or variables with different measurement levels (e.g., continuous subscale scores and categorical item scores; see [16]), and exogenous variables that possibly explain response shift. For an interpretation of the impact of response shift on the assessment of change, it is also possible to calculate SEM-based effect-size indices [12].

## Practical considerations in application of the SEM approach

As with any method, the validity of the SEM method depends on certain methodological and conceptual assumptions. General discussions about the underlying assumptions of SEM (e.g., [11, 17]), and the measurement of latent variables (e.g., [18, 19]) can be found elsewhere. Here, we focus on practical issues that are specifically important for valid application and interpretation of the SEM approach for detecting response shift in HRQL outcomes. Table 2 lists these issues and connects them to the four different steps in the SEM procedure.

**Table 2:** Overview of practical considerations in application of the SEM approach for the detection of response shift

	Decisions to be made	Recommendations
Know your measures		
Step 1: establishing a measurement model	• Choose one of two procedures (i.e., model all measurement occasions simultaneously or separately) to arrive at a longitudinal measurement model • Modify the measurement model when model fit is not adequate, in order to obtain a well-fitting model • Decide which and how many modifications are necessary to obtain a substantively meaningful measurement model	• Specify the measurement model based on the structure of the questionnaire, previous research, and/or theory • In case of unclear or unknown structure, use exploratory analyses and substantive considerations to arrive at an interpretable and well-fitting measurement model • Combine substantive and statistical criteria to guide (re)specification to arrive at the most parsimonious, most reasonable, and best-fitting measurement model
Identification of possible response shift		
Step 2: overall test of response shift Step 3: detection of response shift	• Choose statistical criteria to guide response shift detection • Choose between competing response shifts • Decide when to stop searching for response shift	• Use the overall test of response shift to protect against false detection (i.e., type I error) • When possible, use an iterative procedure (where all possible response shifts are considered one at a time) to identify specific response shift effects • Alternatively, use statistical indices such as modification indices, expected parameter change, inspection of residuals, and/or Wald tests to guide response shift detection • Evaluate each possible response shift statistically (i.e., difference in model fit) and substantively (i.e., interpretation) in order to identify all meaningful effects • For more robust stopping criteria, use overall model-fit evaluation and evaluation of difference in model fit of the measurement model • Use different sequential decision-making practices in order to find confidence in robustness of results
Interpretation of response shift and ‘true’ change		
Step 3: detection of response shift Step 4: assessment of ‘true’ change	• Can the detected violations of invariance of model parameters be interpreted as response shifts? • Is there ‘true’ change? • What is the impact of response shift on the assessment of change?	• Detected effects can be substantively interpreted as response shift using substantive knowledge of the patient group, treatment, or disease trajectory • Compare common factor means across occasions to assess ‘true’ change in the target construct • To understand both ‘true’ change and response shift, consider possible other explanations for the detected effects and include—when available—possible explanatory variables (e.g., coping, adaptation, social comparison) • To understand the impact of response shift on change assessment, evaluate (1) the impact of response shift on observed change in the indicator variable using the decomposition of change [12], and (2) the impact of response shift on ‘true’ change by comparing the common factor means from the final model of Step 3 (while taking into account response shift) with the common factor means from Step 2 (under no response shift)

### Know your measures: establishing an appropriate measurement model

The measurement model specifies the relations between the observed variables and underlying latent factor(s) and thus defines the construct that we intend to measure. With longitudinal data, the measurement model includes the specification of the measurement structure at each measurement occasion and also referred to as the longitudinal measurement model (LMM). To arrive at the LMM (i.e., step 1 of the SEM approach), one can establish an appropriate measurement model for each measurement occasion separately and combine all separate measurement models into a single LMM (cf. [20]). Or, alternatively, one can combine all measurement occasions into a single longitudinal measurement model and establish an appropriate LMM for all measurement occasions simultaneously (cf. [21]). The only requirements of the specified LMM are that the measurement structure is largely the same (e.g., the same number of underlying common factors) across time, and that it has interpretable common factors. In practice of response shift detection, however, differences in the measurement structure are indicative of reconceptualization response shift. Therefore, the measurement structure is often specified to be the same at each occasion. For example, the LMM of our illustrative example of HRQL is specified by using the three-factor model from Fig. 1 at both baseline and follow-up (see Fig. 2).

An appropriate starting point for the specification of a measurement model can be based on the structure of the questionnaire, results from previous research, substantive considerations about the content of the observed measures, exploratory factor analyses, or—more likely—a combination of these approaches. For example, when a HRQL questionnaire is developed based on the idea that the items reflect social, mental, and physical aspects of health, then the measurement model could be specified as a three-factor model, where all items that measure the same domain load on the associated common factor (such as in Fig. 1). However, specification of the measurement model can become more complicated in situations where the dimensional structure of a questionnaire is unclear, or where (items of) different questionnaires are combined (cf. [14]). Moreover, it is often necessary to modify the initially specified measurement model to obtain a well-fitting model. A well-fitting measurement model is necessary, as the measurement model is the baseline model against which all further models (that are used to test for the presence of response shift) will be compared. Thus, the measurement model represents the most parsimonious, the most reasonable or defendable, and the best-fitting model to the data [22].

To evaluate whether the model fit of the measurement model is appropriate (e.g., assessment of overall model fit) and to guide model specification, when the initial model fit is suboptimal or inadequate (e.g., using differences in model fit), one can use statistical criteria. However, evaluation of statistical criteria for (differences in) model fit is complicated by the fact that there exist many different fit indices, with different decision rules that may be more or less appropriate depending on the context of the study. An overview of the most important fit measures and their (dis)advantages are provided in Table 3. As a general recommendation, the researcher could inspect and report several fit indices but should be aware that choice of the specific fit index might depend on the specifics of the data (e.g., sample size), complexity of the model, and/or the hypothesis that is being tested. Detailed discussions on the use of different SEM-based fit indices are provided elsewhere (e.g., see [23–25]).

**Table 3:** An overview of SEM-based model-fit indices for the evaluation of overall goodness of model fit and differences in model fit

Description and interpretation	Advantages	Disadvantages
Fit statistic		
*Overall goodness of model fit*		
Chi-square test		
The chi-square value can be used to test the null hypothesis of ‘exact’ fit (i.e., that the specified model holds exactly in the population), where a significant chi-square value indicates a significant deviation between the model and data (and thus that the model does not hold exactly in the population)	• Has a clear interpretation • Provides a convenient decision rule	• Highly dependent on sample size, i.e., with increasing sample size and equal degrees of freedom the chi-square value increases • Tends to favor highly parameterized (i.e., complex) models
Root-mean-square error of approximation (RMSEA)		
The RMSEA [34, 35] is a measure of approximate fit. According to a generally accepted rule of thumb, an RMSEA value below .08 indicates ‘reasonable’ fit and one below .05 ‘close’ fit [36]	• Confidence intervals are available • Can be used to provide a ‘test of close fit’ • Takes into account model complexity • Less sensitive to sample size	• When N or df is relatively small, the index is expected to be uninformative [37]
Comparative fit index (CFI)		
The CFI [38] gives an indication of model fit based on model comparison (compared to the independence model in which all measured variables are uncorrelated). The CFI ranges from zero to one, and as a general rule of thumb, values above 0.95 are indicative of relatively ‘good’ model fit [24]. The Tucker-Lewis index (TLI [39]; also referred to as the non-normed fit index (NNFI)) is a similar comparative fit index	• Relatively unaffected by sample size • Takes into account model complexity	• Does not provide a test of model fit • Sensitive to the size of the observed covariances
Expected cross-validation Index (ECVI)		
The ECVI [40] is a measure of approximate fit that is especially useful for the comparison of different models for the same data, where the model with the smallest ECVI indicates the model with the best fit. It provides the same ranking of models as the Akaike Information Criterion (AIC; [41]) and so-called “Bayesian” Information Criterion (BIC; [42, 43])	• Confidence intervals are available • Takes into account model complexity	• Does not provide a test of model fit • Cannot be used to evaluate model fit of a single model
*Difference in model fit*		
The chi-square difference test		
The chi-square values of two nested models (i.e., where the second model can be derived from the first model by imposing restrictions on model parameters) can be compared to test the difference in ‘exact’ fit. A significant difference in chi-square values indicates that the less restricted model fits the observed data significantly better than the more restricted model, or in other words, the more restricted model leads to a significant deterioration in model fit	• Has a clear interpretation • Provides a convenient decision rule	• Highly dependent on sample size (see above) • Tends to favor highly parameterized (i.e., complex) models
ECVI difference test		
The difference in ECVI values of two nested models may be used to test the equivalence in approximate model fit, where a value that is significantly larger than zero indicates that the more restricted model has significantly worse approximate fit. Similarly, the difference between two model’s AICs (or BICs) can be used	• Takes into account model complexity	• Stringent evaluation of the performance of the ECVI for the comparison of nested models is lacking
CFI difference		
It has been proposed that the difference between CFI values can be used to evaluate the difference in model fit between two nested models [44]. As a rule of thumb, CFI difference values larger than 0.01 are taken to indicate that the more restricted model should be rejected	• Simple to apply	• Cannot be used to test whether the difference in model fit is significant

Making decisions in model (re-)specification also require substantive considerations (i.e., does a model make sense?). For example, statistical indices may indicate that the largest improvement in fit can be achieved by freeing a factor loading of a physical functioning item on a common factor that measures mental health; such a model specification may not make sense substantively. On the other hand, freeing a residual covariance between indicators that share the same item format may be sensible cf. [26] even though it will not lead to a large improvement in model fit or to a change in interpretation of the common factors. In order to find a substantively reasonable measurement model, it is at least equally—and possibly even more—important to rely on substantive knowledge as on statistical criteria.

### Identification of possible response shift

The mere presence of response shift is evaluated by testing whether the equality restrictions on all model parameters associated with response shift are tenable (i.e., Step 2 of the SEM procedure), representing an ‘omnibus test’ for the presence of response shift. This procedure has also been advocated by others [27] and has been shown to protect against false positives [28]. However, if there is evidence of the presence of response shift, how does one then accurately locate which observed variable is affected by which type of response shift?

The search for response shift (i.e., step 3 of the SEM approach) requires exploratory model fitting or re-specification, which is referred to as the ‘specification search.’ The specification search can be guided using statistical criteria, such as modification indices, expected parameter changes, Wald tests, inspection of residuals, or differences in model fit [29]. In order to correctly identify the change in model parameters, it has been recommended to use an iterative procedure [30], where all model parameters associated with response shift are freed one at a time, and the freely estimated parameter that shows the largest improvement in model fit is incorporated in the model. However, it may be that two different model modifications lead to equivalent improvement in model fit. A decision on which model modification to prioritize can, therefore, not be based on statistical criteria alone. Given the dependence of sequential model re-specification, freeing one model parameter may render freeing the other model parameter unnecessary, i.e., a change to the model can affect other parts of the model too. It may therefore be possible that alternative series of model re-specifications lead to different results. For example, in the search for response shift in our illustrative example of HRQL, it may be that freeing the intercept value of either ‘family relations’ or ‘friendships’ (both indicators of social health; see Fig. 1) would lead to an equivalent improvement in model fit, but that freeing one would render freeing the other intercept unnecessary. One thus needs other—substantive—reasons to decide on which response shift effect to include in the model. It may be, for example that recalibration of ‘family relations’ is much more plausible given the type of catalyst (e.g., type of disease or treatment) or prevalence of married patients/marital status in the study population.

Instead of strictly adhering to a procedure where only the modification that leads to the largest model-fit improvement is considered, it may be important to follow different sequences in model re-specification—i.e., choose different modifications that lead to different but more-or-less equivalent model-fit improvement—to investigate whether and to what extent these different sequential decisions lead to different results. This will allow the researcher to see whether detection of response shift is dependent on sequential decision-making practices and to choose among possible differences in these sequences based on a combination of both statistical and substantive considerations. It is this repeated back-and-forth specification search in which one can find confidence in the robustness of results or, alternatively, find that a confident conclusion about the number and types of response shift is not warranted. Clearly, these sequential decision-making practices thus require subjective judgment, and different researchers may make different decisions. This is a necessary consequence of ensuring interpretability of findings. For example, it may be that in different sequences of response shift investigation for our illustrative example of HRQL, the difference in intercepts of ‘family relations’ re-occurs frequently, while the difference in intercepts of ‘friendships’ only occurs sporadically. Such a pattern of results may help to decide between different modifications that lead to similar improvements in model fit.

The specification search for possible response shift effects also requires a decision on when to stop searching. The aim of the specification search is to identify all possible response shift effects (i.e., identify all true positives). Meanwhile, however, one wants to prevent the identification of trivial differences in model parameters across time as being of substantive interest (i.e., identification of false positives, or type 1 errors). In addition to the improvement in model fit for freeing individual parameters, one can rely on the difference in model fit between the measurement model and the model that includes all identified response shift effects. When the overall difference in fit between these models is not significant, this may be taken as an indication that freeing additional model parameters is no longer necessary. Also, one can use the overall model fit of the model to judge whether the model that includes response shift is tenable. These model-fit evaluations may provide more robust stopping criteria. However, it has also been argued that in order to adequately identify all response shift effects, it may be necessary to continue the specification search, even when the established model already shows adequate model fit [31]. Therefore, model-fit criteria should be used in combination with substantive criteria with regard to the (possible) response shifts. For example, it may be that freeing an additional model parameter will lead to a small, non-significant improvement in model fit, but that the associated response shift has a clear interpretation. For example, when in our illustrative example of HRQL, there is an a-priori hypothesis about the occurrence of reprioritization response shift of ‘nausea’ (see Table 1), it may be informative to report on a small but non-significant effect. As a researcher, one has to find a balance between the goodness of fit and the interpretability of the model. Again, subjective judgment is needed to ensure meaningfulness of the results.

### Interpretation of detected response shift and ‘true’ change

With SEM, we do not look at response shifts directly, but at the effects, these response shifts have on the measurement of change in HRQL. This allows us to describe *what* occurs (i.e., patterns of different types of change), but it does not imply that we also know *how* it occurs (i.e., what the causes are of the identified response shift). For the substantive interpretation of change, it is therefore important to provide an interpretation and possible explanation of detected response shift. For example, imagine that in our illustrative example, recalibration was detected in the indicator ‘pain’ of physical health, where patients showed a larger decrease in pain as compared to the other indicators of physical health. A possible explanation for this result may be that patients adapted to the experience of pain and therefore rated their pain to be lower at follow-up, even though their actual experience of pain did not change (or changed to a lesser degree), i.e., recalibration response shift (see Table 1). It may also be that patients received treatment or medication that reduced their experienced level of pain. However, one could argue that only the first interpretation coincides with what Sprangers and Schwartz [4] describe as recalibration response shift. The SEM approach for the detection of response shift does not make such substantive distinctions. Therefore, substantive interpretation of detected response shift is of paramount importance; it is needed both to clarify what is taken as evidence of response shift and to exclude, or make less likely, alternative explanations.

The interpretation of detected response shifts can be based on substantive knowledge of the patient group, the treatment, or disease trajectory. In addition, it is possible to include operationalizations of potential explanations of response shift in the SEM model. If measures of antecedents (e.g., sociodemographic or personality characteristics) or mechanisms (e.g., coping strategies, social comparison) are available, they can be incorporated in the model as possible explanatory variables for response shift effects cf. [32]. For example, in order to investigate the role of appraisal processes (following [7]) for the detected recalibration response shift of pain as described above, one could include a direct measure of appraisal in the model and investigate the effect of appraisal on the (change in) scores of the indicator ‘pain.’ Such investigations will help to substantiate whether and how the detected response shifts are influenced by individuals’ cognitive changes in standards, values, or conceptualizations. As such, substantive interpretation and explanation of response shift are necessary to understand both the mechanisms of response shift, and how it affects change in the construct that we intend to measure (i.e., HRQL), which in turn will help to better understand patients’ perceived health trajectories.

Finally, the (clinical) relevance of occurrences of response shift can be evaluated by calculating the impact of response shift on the assessment of change. First, the decomposition of change [12] can be used to interpret the impact of response shift on change in the observed variables (e.g., change in item scores). The decomposition entails that observed change is decomposed into so-called ‘true’ change (i.e., change due to change in the underlying target construct) and change due to response shift. Second, the impact of response shift on ‘true’ change in the underlying target construct (e.g., HRQL) can be evaluated by comparing estimates of change before and after taking into account response shifts. SEM-based effect-size indices can help to interpret the magnitude of the impact on change assessment [12]. This is important because substantial and interpretable response shifts do not always exert a considerable impact on ‘true’ change. For example, it may be that the detected recalibration response shift in the indictor ‘pain’ is statistically significant, interpretable (see above), and has substantial impact on the observed change in pain. At the same time, it may be that ‘true’ change in physical health is not influenced by the detected response shift. Then, the detected recalibration response shift has no impact on the interpretation of change in HRQL. Still, the occurrence (and investigation) of response shift is insightful because it shows how change in the target construct is (differentially) related to change in the observed measures. Both types of information regarding the impact of response shift on change assessment can thus be used to better interpret the findings from response shift investigations.

## Conclusion

In the current paper, we discuss practical issues that are important for researchers who want to apply SEM for the assessment of change and detection of response shift. We provide general recommendations that can be used for all applications, while acknowledging that decisions are made on a case-by-case basis and require the substantive issues at stake. We wish to emphasize the importance of taking into account substantive considerations in addition to statistical information to guide the sequential decision-making practices. These decisions require subjective judgment and are needed for any statistical modeling procedure to ensure interpretability of findings. Moreover, for a meaningful interpretation of change, it is important to try to substantiate the linkage between detected response shift and patients’ perceived health trajectories, e.g., by using substantive knowledge or direct measures of possible explanatory variables. With the recommendations provided in this paper, we aim to stimulate the appropriate application and interpretation of SEM for the investigation of response shift and assessment of change in PROs and thus improve the scientific stringency of the field. As sound statistical techniques can contribute to a better understanding of patients’ perceived health trajectories, this will ultimately improve the evaluation and interpretation of the effectiveness of health-care interventions and thus improve the quality of patients’ lives.

## Supplementary Information

Below is the link to the electronic supplementary material.

Supplementary file1 (PDF 1135 KB)

Supplementary file1 (R 8 KB)

Supplementary file1 (TXT 7 KB)
