# Supplementary material for: Using structural equation modeling to investigate change and response shift in patient-reported outcomes: practical considerations and recommendations
Source: Qual Life Res. 2021 Feb 7;30(5):1293–304. doi: 10.1007/s11136-020-02742-9 (PMC8068637; doi:10.1007/s11136-020-02742-9)
Supplement: Supplementary file 1 — Supplementary file1 (PDF 1135 KB) [file 11136_2020_2742_MOESM1_ESM.pdf]

Appendix A. Explanation of structural equation modeling (SEM) parameters

Suppose we have patients' scores on nine different observed item scores from a health-related quality of life (HRQL) questionnaire that measures physical (i.e. 'nausea', 'pain', and 'fatigue'), mental ('anxiety', 'sadness', and 'happiness') and social ('family relations', 'friend relations', and 'work relations') aspects of health. We use a (three-) factor model to describe the relationships between the observed variables (i.e. item scores) (see also Figure 1). Within the structural equation modeling (SEM) framework, the variances and covariances ($\mathbf{\Sigma}$, 'Sigma') and means ($\boldsymbol{\mu}$, 'mu') of the observed variables (X) are given by:

$$\text{Cov}(X,X') = \mathbf{\Sigma} = \mathbf{\Lambda}\ \mathbf{\Phi}\ \mathbf{\Lambda}' + \mathbf{\Theta},$$

and:

$$\text{Mean}(X) = \boldsymbol{\mu} = \boldsymbol{\tau} + \mathbf{\Lambda}\ \boldsymbol{\kappa},$$

where:

$\mathbf{\Sigma}$ ('Sigma') is a 9x9 symmetric matrix that contains the observed variances and covariances of the indicator variables, i.e. the observed variances and covariances of the nine item scores.

$\mathbf{\Lambda}$ ('Lambda') is a 9x3 matrix of common factor loadings, that describes which of the nine observed indicator (i.e. item scores) measures which of the three common factors (i.e. the three aspects of health).

$\mathbf{\Phi}$ ('Phi') is a 3x3 symmetric matrix of common factor variances and covariances, that describes the variability and interrelations between the three different aspects of health.

$\mathbf{\Theta}$ ('Theta') is a 9x9 diagonal matrix of residual variances, that describe the variances of the nine observed indicators that cannot be explained by the underlying common factors.

$\boldsymbol{\mu}$ ('mu') is a 9x1 vector that contains the observed means of the indicator variables, i.e. the means of the nine item scores.

$\boldsymbol{\tau}$ ('tau') is a 9x1 vector that contains the intercepts values for each indicator, i.e. nine intercept values for the nine observed item scores.

$\boldsymbol{\kappa}$ ('kappa') is a 3x1 vector that contains the common factor means, i.e. the means of the three underlying aspects of health.

$$
\boldsymbol{\Sigma} = \begin{bmatrix}
\sigma_1^2 &&&&&&&& \\
\sigma_{2,1} & \sigma_2^2 &&&&&&& \\
\sigma_{3,1} & \sigma_{3,2} & \sigma_3^2 &&&&&& \\
\sigma_{4,1} & \sigma_{4,2} & \sigma_{4,3} & \sigma_4^2 &&&&& \\
\sigma_{5,1} & \sigma_{5,2} & \sigma_{5,3} & \sigma_{5,4} & \sigma_5^2 &&&& \\
\sigma_{6,1} & \sigma_{6,2} & \sigma_{6,3} & \sigma_{6,4} & \sigma_{6,5} & \sigma_6^2 &&& \\
\sigma_{7,1} & \sigma_{7,2} & \sigma_{7,3} & \sigma_{7,4} & \sigma_{7,5} & \sigma_{7,6} & \sigma_7^2 && \\
\sigma_{8,1} & \sigma_{8,2} & \sigma_{8,3} & \sigma_{8,4} & \sigma_{8,5} & \sigma_{8,6} & \sigma_{8,7} & \sigma_8^2 & \\
\sigma_{9,1} & \sigma_{9,2} & \sigma_{9,3} & \sigma_{9,4} & \sigma_{9,5} & \sigma_{9,6} & \sigma_{9,7} & \sigma_{9,8} & \sigma_9^2
\end{bmatrix},
$$

where σ_1^2 contains the observed variance of X_1 (i.e. item scores of 'nausea') and $\sigma_{1,2}$ contains the observed covariance between X_1 and X_2 (i.e. covariance between item scores on 'nausea' and 'pain').

$$
\boldsymbol{\Lambda} = \begin{bmatrix}
\lambda_{1,1} && \\
\lambda_{2,1} && \\
\lambda_{3,1} && \\
& \lambda_{4,2} & \\
& \lambda_{5,2} & \\
& \lambda_{6,2} & \\
&& \lambda_{7,3} \\
&& \lambda_{8,3} \\
&& \lambda_{9,3}
\end{bmatrix},
$$

where $\lambda_{1,1}$ contains the factor loading of X_1 ('nausea') on the first common factor (physical health), and $\lambda_{7,3}$ contains the factor loading of X_7 ('friend relations') on the third common factor (social health).

$$
\boldsymbol{\Phi} = \begin{bmatrix}
\varphi_1^2 && \\
\varphi_{2,1} & \varphi_2^2 & \\
\varphi_{3,1} & \varphi_{3,2} & \varphi_3^2
\end{bmatrix},
$$

where φ_1^2 contains the variance of the first common factor (physical health), and $\varphi_{1,2}$ contains the covariance between the first two common factors (physical and mental health).

$$
\boldsymbol{\Theta} = \begin{bmatrix}
\theta_1^2 &&&&&&&& \\
& \theta_2^2 &&&&&&& \\
&& \theta_3^2 &&&&&& \\
&&& \theta_4^2 &&&&& \\
&&&& \theta_5^2 &&&& \\
&&&&& \theta_6^2 &&& \\
&&&&&& \theta_7^2 && \\
&&&&&&& \theta_8^2 & \\
&&&&&&&& \theta_9^2
\end{bmatrix},
$$

where θ_4^2 contains the residual variance of observed indicator X_4 ('anxiety').

$$\boldsymbol{\mu} = \begin{bmatrix} \mu_1 \\ \mu_2 \\ \mu_3 \\ \mu_4 \\ \mu_5 \\ \mu_6 \\ \mu_7 \\ \mu_8 \\ \mu_9 \end{bmatrix},$$

where μ_1 contains the mean of X_1 (i.e. the mean of the observed item scores on 'nausea').

$$\boldsymbol{\tau} = \begin{bmatrix} \tau_1 \\ \tau_2 \\ \tau_3 \\ \tau_4 \\ \tau_5 \\ \tau_6 \\ \tau_7 \\ \tau_8 \\ \tau_9 \end{bmatrix},$$

where $\boldsymbol{\tau}_1$ contains the intercept value of X_1.

$$\boldsymbol{\kappa} = \begin{bmatrix} \kappa_1 \\ \kappa_2 \\ \kappa_3 \end{bmatrix},$$

where κ_1 contains the mean of the first common factor (i.e. physical health).

Cov(X,X') = $\mathbf{\Sigma} = \mathbf{\Lambda}\ \mathbf{\Phi}\ \mathbf{\Lambda'} + \mathbf{\Theta}$

$$\begin{bmatrix} \sigma_1^2 &&&&&&&& \\ \sigma_{2,1} & \sigma_2^2 &&&&&&& \\ \sigma_{3,1} & \sigma_{3,2} & \sigma_3^2 &&&&&& \\ \sigma_{4,1} & \sigma_{4,2} & \sigma_{4,3} & \sigma_4^2 &&&&& \\ \sigma_{5,1} & \sigma_{5,2} & \sigma_{5,3} & \sigma_{5,4} & \sigma_5^2 &&&& \\ \sigma_{6,1} & \sigma_{6,2} & \sigma_{6,3} & \sigma_{6,4} & \sigma_{6,5} & \sigma_6^2 &&& \\ \sigma_{7,1} & \sigma_{7,2} & \sigma_{7,3} & \sigma_{7,4} & \sigma_{7,5} & \sigma_{7,6} & \sigma_7^2 && \\ \sigma_{8,1} & \sigma_{8,2} & \sigma_{8,3} & \sigma_{8,4} & \sigma_{8,5} & \sigma_{8,6} & \sigma_{8,7} & \sigma_8^2 & \\ \sigma_{9,1} & \sigma_{9,2} & \sigma_{9,3} & \sigma_{9,4} & \sigma_{9,5} & \sigma_{9,6} & \sigma_{9,7} & \sigma_{9,8} & \sigma_9^2 \end{bmatrix} =$$

$$\begin{bmatrix} \lambda_{1,1} && \\ \lambda_{2,1} && \\ \lambda_{3,1} && \\ & \lambda_{4,2} & \\ & \lambda_{5,2} & \\ & \lambda_{6,2} & \\ && \lambda_{7,3} \\ && \lambda_{8,3} \\ && \lambda_{9,3} \end{bmatrix} \begin{bmatrix} \varphi_1^2 && \\ \varphi_{2,1} & \varphi_2^2 & \\ \varphi_{3,1} & \varphi_{3,2} & \varphi_3^2 \end{bmatrix} \begin{bmatrix} \lambda_{1,1} & \lambda_{2,1} & \lambda_{3,1} &&&&&& \\ &&& \lambda_{4,2} & \lambda_{5,2} & \lambda_{6,2} &&& \\ &&&&&& \lambda_{7,3} & \lambda_{8,3} & \lambda_{9,3} \end{bmatrix} + \begin{bmatrix} \theta_1^2 &&&&&&&& \\ & \theta_2^2 &&&&&&& \\ && \theta_3^2 &&&&&& \\ &&& \theta_4^2 &&&&& \\ &&&& \theta_5^2 &&&& \\ &&&&& \theta_6^2 &&& \\ &&&&&& \theta_7^2 && \\ &&&&&&& \theta_8^2 & \\ &&&&&&&& \theta_9^2 \end{bmatrix}$$

Mean(X) = $\boldsymbol{\mu} = \boldsymbol{\tau} + \boldsymbol{\Lambda}\ \boldsymbol{\kappa}$

$$\begin{bmatrix}\mu_1\\\mu_2\\\mu_3\\\mu_4\\\mu_5\\\mu_6\\\mu_7\\\mu_8\\\mu_9\end{bmatrix} = \begin{bmatrix}\tau_1\\\tau_2\\\tau_3\\\tau_4\\\tau_5\\\tau_6\\\tau_7\\\tau_8\\\tau_9\end{bmatrix} + \begin{bmatrix}\lambda_{1,1} & & \\ \lambda_{2,1} & & \\ \lambda_{3,1} & & \\ & \lambda_{4,2} & \\ & \lambda_{5,2} & \\ & \lambda_{6,2} & \\ & & \lambda_{7,3} \\ & & \lambda_{8,3} \\ & & \lambda_{9,3}\end{bmatrix} \begin{bmatrix}\kappa_1\\\kappa_2\\\kappa_3\end{bmatrix}$$

Appendix B. Explanation of full matrices used in the structural equation modeling (SEM) response shift detection method applied to two measurement occasions

We use the illustrative example of the health-related quality of life (HRQL) questionnaire with nine observed indicators that measure physical, mental and social aspects of health (see Figure 1). The three-factor model is applied at two occasions, i.e. baseline (T1) and follow-up (T2). The variances and covariances (**Σ**, 'Sigma') and means (**μ**, 'mu') of the observed variables (*X*) are given by:

$$\mathrm{Cov}(X,X') = \mathbf{\Sigma} = \mathbf{\Lambda}\ \mathbf{\Phi}\ \mathbf{\Lambda}' + \mathbf{\Theta},$$

and:

$$\mathrm{Mean}(X) = \boldsymbol{\mu} = \boldsymbol{\tau} + \mathbf{\Lambda}\ \boldsymbol{\kappa}.$$

As all matrices now contain parameters of the same measurements at two occasions, they can be rewritten into block matrices that contain the parameters of each occasion, and the longitudinal relations, as follows:

$$\begin{bmatrix} \mathbf{\Sigma}_{T1} & \mathbf{\Sigma}_{T1,T2} \\ \mathbf{\Sigma}_{T2,T1} & \mathbf{\Sigma}_{T2} \end{bmatrix} = \begin{bmatrix} \mathbf{\Lambda}_{T1} & \mathbf{0} \\ \mathbf{0} & \mathbf{\Lambda}_{T2} \end{bmatrix} \begin{bmatrix} \mathbf{\Phi}_{T1} & \mathbf{\Phi}_{T1,T2} \\ \mathbf{\Phi}_{T2,T1} & \mathbf{\Phi}_{T2} \end{bmatrix} \begin{bmatrix} \mathbf{\Lambda}_{T1} & \mathbf{0} \\ \mathbf{0} & \mathbf{\Lambda}_{T2} \end{bmatrix}' + \begin{bmatrix} \mathbf{\Theta}_{T1} & \mathbf{\Theta}_{T1,T2} \\ \mathbf{\Theta}_{T2,T1} & \mathbf{\Theta}_{T2} \end{bmatrix}$$

and:

$$\begin{bmatrix} \boldsymbol{\mu}_{T1} \\ \boldsymbol{\mu}_{T2} \end{bmatrix} = \begin{bmatrix} \boldsymbol{\tau}_{T1} \\ \boldsymbol{\tau}_{T2} \end{bmatrix} + \begin{bmatrix} \mathbf{\Lambda}_{T1} & \mathbf{0} \\ \mathbf{0} & \mathbf{\Lambda}_{T2} \end{bmatrix} \begin{bmatrix} \boldsymbol{\kappa}_{T1} \\ \boldsymbol{\kappa}_{T2} \end{bmatrix},$$

where:

Σ ('Sigma') is a 18x18 block matrix that contains the observed variances and covariances of the indicator variables, i.e. the observed variances and covariances of the nine item scores at two measurement occasions; $\mathbf{\Sigma}_{T1}$ and $\mathbf{\Sigma}_{T1}$ are 9x9 symmetric matrices that contain the observed variance and covariances at baseline and follow-up respectively, and $\mathbf{\Sigma}_{T2,T1}$ is a 9x9 matrix that contains the longitudinal relations between the observed variables.

Λ ('Lambda') is a 18x6 block matrix of common factor loadings, that describes which of the nine observed indicator (i.e. item scores) measures which of the three common factors (i.e. the three aspects of health), at both occasions; $\mathbf{\Lambda}_{T1}$ and $\mathbf{\Lambda}_{T2}$ are 9x3 matrices that contain the common factor loadings at baseline and follow-up respectively.

Φ ('Phi') is a 6x6 block matrix of common factor variances and covariances, that describes the variability and interrelations between the three different aspects of health at both occasions; $\mathbf{\Phi}_{T1}$ and $\mathbf{\Phi}_{T2}$ are 3x3 symmetric matrices that contain the common factor variance

and covariances at baseline and follow-up respectively, and $\mathbf{\Phi}_{T2,T1}$ is a 3x3 symmetric matrix that contains the longitudinal relations between the common factors.

$\mathbf{\Theta}$ ('Theta') is a 18x18 block matrix of residual variances and covariances, that describe the variances and covariances of the eighteen observed indicators that cannot be explained by the underlying common factors. $\mathbf{\Theta}_{T1}$ and $\mathbf{\Theta}_{T2}$ are 9x9 diagonal matrices that contain the residual factor variances at baseline and follow-up respectively, and $\mathbf{\Theta}_{T2,T1}$ is a 9x9 diagonal matrix that contains the longitudinal relations between the residual factors, i.e. the residual factor covariances between the same indicators measured at both occasions.

$\boldsymbol{\mu}$ ('mu') is a 18x1 stacked vector that contains the observed means of the indicator variables, i.e. the means of the nine item scores at two measurement occasions; $\boldsymbol{\mu}_{T1}$ and $\boldsymbol{\mu}_{T2}$ are 9x1 vectors that contain the observed means at baseline and follow-up respectively.

$\boldsymbol{\tau}$ ('tau') is a 18x1 stacked vector that contains the intercepts values for each indicator, i.e. eighteen intercept values for the nine observed item scores at both measurement occasions; $\boldsymbol{\tau}_{T1}$ and $\boldsymbol{\tau}_{T2}$ are 9x1 vectors that contain nine intercept values of the indicator variables at baseline and follow-up respectively.

$\boldsymbol{\kappa}$ ('kappa') is a 6x1 stacked vector that contains the common factor means, i.e. the means of the three underlying aspects of health, at both occasions; $\boldsymbol{\kappa}_{T1}$ and $\boldsymbol{\kappa}_{T2}$ are 3x1 vectors that contain the common factor means at baseline and follow-up respectively.

$$\boldsymbol{\Sigma} = \begin{bmatrix} \boldsymbol{\Sigma}_{\mathbf{T1}} & \boldsymbol{\Sigma}_{\mathbf{T1,T2}} \\ \boldsymbol{\Sigma}_{\mathbf{T2,T1}} & \boldsymbol{\Sigma}_{\mathbf{T2}} \end{bmatrix},$$

where:

$$\boldsymbol{\Sigma}_{\mathbf{T1}} = \begin{bmatrix} \sigma^2_{1_{T1}} & & & & & & & & \\ \sigma_{2_{T1},1_{T1}} & \sigma^2_{2_{T1}} & & & & & & & \\ \sigma_{3_{T1},1_{T1}} & \sigma_{3_{T1},2_{T1}} & \sigma^2_{3_{T1}} & & & & & & \\ \sigma_{4_{T1},1_{T1}} & \sigma_{4_{T1},2_{T1}} & \sigma_{4_{T1},3_{T1}} & \sigma^2_{4_{T1}} & & & & & \\ \sigma_{5_{T1},1_{T1}} & \sigma_{5_{T1},2_{T1}} & \sigma_{5_{T1},3_{T1}} & \sigma_{5_{T1},4_{T1}} & \sigma^2_{5_{T1}} & & & & \\ \sigma_{6_{T1},1_{T1}} & \sigma_{6_{T1},2_{T1}} & \sigma_{6_{T1},3_{T1}} & \sigma_{6_{T1},4_{T1}} & \sigma_{6_{T1},5_{T1}} & \sigma^2_{6_{T1}} & & & \\ \sigma_{7_{T1},1_{T1}} & \sigma_{7_{T1},2_{T1}} & \sigma_{7_{T1},3_{T1}} & \sigma_{7_{T1},4_{T1}} & \sigma_{7_{T1},5_{T1}} & \sigma_{7_{T1},6_{T1}} & \sigma^2_{7_{T1}} & & \\ \sigma_{8_{T1},1_{T1}} & \sigma_{8_{T1},2_{T1}} & \sigma_{8_{T1},3_{T1}} & \sigma_{8_{T1},4_{T1}} & \sigma_{8_{T1},5_{T1}} & \sigma_{8_{T1},6_{T1}} & \sigma_{8_{T1},7_{T1}} & \sigma^2_{8_{T1}} & \\ \sigma_{9_{T1},1_{T1}} & \sigma_{9_{T1},2_{T1}} & \sigma_{9_{T1},3_{T1}} & \sigma_{9_{T1},4_{T1}} & \sigma_{9_{T1},5_{T1}} & \sigma_{9_{T1},6_{T1}} & \sigma_{9_{T1},8_{T1}} & \sigma_{9_{T1},8_{T1}} & \sigma^2_{9_{T1}} \end{bmatrix},$$

where $\sigma^2_{1_{T1}}$ contains the observed variance of X_1 (i.e. item scores of 'nausea') at baseline occasions (T1), and $\sigma_{2_{T1},1_{T1}}$ contains the observed covariance between X_1 and X_2 at baseline occasion (i.e. the covariance between item scores on 'nausea' and 'pain'). In the same way $\boldsymbol{\Sigma}_{\mathbf{T2}}$ contains the observed variance and covariances of the observed variables at follow-up occasions (T2).

$$\boldsymbol{\Sigma}_{\mathbf{T2,T1}} = \begin{bmatrix} \sigma_{1_{T2},1_{T1}} & \sigma_{1_{T2},2_{T1}} & \sigma_{1_{T2},3_{T1}} & \sigma_{1_{T2},4_{T1}} & \sigma_{1_{T2},5_{T1}} & \sigma_{1_{T2},6_{T1}} & \sigma_{1_{T2},7_{T1}} & \sigma_{1_{T2},8_{T1}} & \sigma_{1_{T2},9_{T1}} \\ \sigma_{2_{T2},1_{T1}} & \sigma_{2_{T2},2_{T1}} & \sigma_{2_{T2},3_{T1}} & \sigma_{2_{T2},4_{T1}} & \sigma_{2_{T2},5_{T1}} & \sigma_{2_{T2},6_{T1}} & \sigma_{2_{T2},7_{T1}} & \sigma_{2_{T2},8_{T1}} & \sigma_{2_{T2},9_{T1}} \\ \sigma_{3_{T2},1_{T1}} & \sigma_{3_{T2},2_{T1}} & \sigma_{3_{T2},3_{T1}} & \sigma_{3_{T2},4_{T1}} & \sigma_{3_{T2},5_{T1}} & \sigma_{3_{T2},6_{T1}} & \sigma_{3_{T2},7_{T1}} & \sigma_{3_{T2},8_{T1}} & \sigma_{3_{T2},9_{T1}} \\ \sigma_{4_{T2},1_{T1}} & \sigma_{4_{T2},2_{T1}} & \sigma_{4_{T2},3_{T1}} & \sigma_{4_{T2},4_{T1}} & \sigma_{4_{T2},5_{T1}} & \sigma_{4_{T2},6_{T1}} & \sigma_{4_{T2},7_{T1}} & \sigma_{4_{T2},8_{T1}} & \sigma_{4_{T2},9_{T1}} \\ \sigma_{5_{T2},1_{T1}} & \sigma_{5_{T2},2_{T1}} & \sigma_{5_{T2},3_{T1}} & \sigma_{5_{T2},4_{T1}} & \sigma_{5_{T2},5_{T1}} & \sigma_{5_{T2},6_{T1}} & \sigma_{5_{T2},7_{T1}} & \sigma_{5_{T2},8_{T1}} & \sigma_{5_{T2},9_{T1}} \\ \sigma_{6_{T2},1_{T1}} & \sigma_{6_{T2},2_{T1}} & \sigma_{6_{T2},3_{T1}} & \sigma_{6_{T2},4_{T1}} & \sigma_{6_{T2},5_{T1}} & \sigma_{6_{T2},6_{T1}} & \sigma_{6_{T2},7_{T1}} & \sigma_{6_{T2},8_{T1}} & \sigma_{6_{T2},9_{T1}} \\ \sigma_{7_{T2},1_{T1}} & \sigma_{7_{T2},2_{T1}} & \sigma_{7_{T2},3_{T1}} & \sigma_{7_{T2},4_{T1}} & \sigma_{7_{T2},5_{T1}} & \sigma_{7_{T2},6_{T1}} & \sigma_{7_{T2},7_{T1}} & \sigma_{7_{T2},8_{T1}} & \sigma_{7_{T2},9_{T1}} \\ \sigma_{8_{T2},1_{T1}} & \sigma_{8_{T2},2_{T1}} & \sigma_{8_{T2},3_{T1}} & \sigma_{8_{T2},4_{T1}} & \sigma_{8_{T2},5_{T1}} & \sigma_{8_{T2},6_{T1}} & \sigma_{8_{T2},7_{T1}} & \sigma_{8_{T2},8_{T1}} & \sigma_{8_{T2},9_{T1}} \\ \sigma_{9_{T2},1_{T1}} & \sigma_{9_{T2},2_{T1}} & \sigma_{9_{T2},3_{T1}} & \sigma_{9_{T2},4_{T1}} & \sigma_{9_{T2},5_{T1}} & \sigma_{9_{T2},6_{T1}} & \sigma_{9_{T2},7_{T1}} & \sigma_{9_{T2},8_{T1}} & \sigma_{9_{T2},9_{T1}} \end{bmatrix},$$

where $\sigma_{4_{T2},1_{T1}}$ contains the covariance between X_4 ('nausea') at follow-up (T2) and X_1 ('anxiety') at baseline (T1), whereas $\sigma_{4_{T1},1_{T2}}$ contains the covariance between X_4 at baseline and X_1 at follow-up occasions. The full $\boldsymbol{\Sigma}$ matrix is symmetric as $\boldsymbol{\Sigma}_{\mathbf{T2,T1}}$ equals $\boldsymbol{\Sigma}_{\mathbf{T2,T1}}$ (e.g. the observed covariance $\sigma_{1_{T2},1_{T1}}$ equals $\sigma_{1_{T1},1_{T2}}$).

$$\mathbf{\Lambda} = \begin{bmatrix} \mathbf{\Lambda_{T1}} & \mathbf{0} \\ \mathbf{0} & \mathbf{\Lambda_{T2}} \end{bmatrix},$$

where:

$$\mathbf{\Lambda_{T1}} = \begin{bmatrix} \lambda_{1_{T1},1_{T1}} & & \\ \lambda_{2_{T1},1_{T1}} & & \\ \lambda_{3_{T1},1_{T1}} & & \\ & \lambda_{4_{T1},2_{T1}} & \\ & \lambda_{5_{T1},2_{T1}} & \\ & \lambda_{6_{T1},2_{T1}} & \\ & & \lambda_{5_{T1},3_{T1}} \\ & & \lambda_{6_{T1},3_{T1}} \\ & & \lambda_{7_{T1},3_{T1}} \end{bmatrix},$$

where $\lambda_{1_{T1},1_{T1}}$ contains the factor loading of X_1 ('nausea') on the first common factor (physical health) at baseline occasions (T1), and $\lambda_{7_{T1},3_{T1}}$ contains the factor loading of X_7 ('friend relations') on the third common factor (social health) at baseline occasion. In the same way, $\mathbf{\Lambda_{T2}}$ contains the factor loadings of the nine observed indicators at follow-up occasion (T2). Because the measurements at baseline occasion do not load on the common factors at follow-up occasion, and vice-versa, the off-diagonal block elements of $\mathbf{\Lambda}$ contain only zeros.

$$\mathbf{\Phi} = \begin{bmatrix} \mathbf{\Phi_{T1}} & \mathbf{\Phi_{T1,T2}} \\ \mathbf{\Phi_{T2,T1}} & \mathbf{\Phi_{T2}} \end{bmatrix},$$

where:

$$\mathbf{\Phi_{T1}} = \begin{bmatrix} \varphi^2_{1_{T1}} & & \\ \varphi_{2_{T1},1_{T1}} & \varphi^2_{2_{T1}} & \\ \varphi_{3_{T1},1_{T1}} & \varphi_{3_{T1},2_{T1}} & \varphi^2_{3_{T1}} \end{bmatrix},$$

where $\varphi^2_{1_{T1}}$ contains the variance of the first common factor (physical health) at baseline occasion (T1), and $\varphi_{2_{T1},1_{T1}}$ contains the covariance between the first two common factors (physical and mental health) at baseline occasion. In the same way $\mathbf{\Phi_{T2}}$ contains the variances and covariances of the common factors at follow-up occasion (T2).

$$\mathbf{\Phi_{T2,T1}} = \begin{bmatrix} \varphi_{1_{T2},1_{T1}} & \varphi_{1_{T2},2_{T1}} & \varphi_{1_{T2},3_{T1}} \\ \varphi_{2_{T2},1_{T1}} & \varphi_{2_{T2},2_{T1}} & \varphi_{2_{T2},3_{T1}} \\ \varphi_{3_{T2},1_{T1}} & \varphi_{3_{T2},2_{T1}} & \varphi_{3_{T2},3_{T1}} \end{bmatrix},$$

where $\varphi_{2_{T2},1_{T1}}$ contains the covariance between the second common factor (mental health) at follow-up (T2) and the first common factor (physical health) at baseline (T1), whereas $\varphi_{1_{T2},2_{T1}}$ contains the covariance between the second common factor at baseline and the first common factor at follow-up. The full $\mathbf{\Phi}$ matrix is symmetric as $\mathbf{\Phi_{T2,T1}} == \mathbf{\Phi_{T2,T1}}$ (e.g. the common factor covariance $\varphi_{1_{T2},1_{T1}}$ equals $\varphi_{1_{T1},1_{T2}}$).

$$\mathbf{\Theta} = \begin{bmatrix} \mathbf{\Theta_{T1}} & \mathbf{\Theta_{T1,T2}} \\ \mathbf{\Theta_{T2,T1}} & \mathbf{\Theta_{T2}} \end{bmatrix},$$

where:

$$\mathbf{\Theta_{T1}} = \begin{bmatrix} \theta_{1_{T1}}^2 & & & & & & & & \\ & \theta_{2_{T1}}^2 & & & & & & & \\ & & \theta_{3_{T1}}^2 & & & & & & \\ & & & \theta_{4_{T1}}^2 & & & & & \\ & & & & \theta_{5_{T1}}^2 & & & & \\ & & & & & \theta_{6_{T1}}^2 & & & \\ & & & & & & \theta_{7_{T1}}^2 & & \\ & & & & & & & \theta_{8_{T1}}^2 & \\ & & & & & & & & \theta_{9_{T1}}^2 \end{bmatrix},$$

where $\theta_{4_{T1}}^2$ contains the residual variance of observed indicator X_4 ('anxiety') at baseline occasion (T1). In the same way $\mathbf{\Theta_{T2}}$ contains the residual variances of the nine observed indicator at follow-up occasion (T2).

$$\mathbf{\Theta_{T2,T1}} = \begin{bmatrix} \theta_{1_{T2},1_{T1}} & & & & & & & & \\ & \theta_{2_{T2},2_{T1}} & & & & & & & \\ & & \theta_{3_{T2},3_{T1}} & & & & & & \\ & & & \theta_{4_{T2},4_{T1}} & & & & & \\ & & & & \theta_{5_{T2},5_{T1}} & & & & \\ & & & & & \theta_{6_{T2},6_{T1}} & & & \\ & & & & & & \theta_{7_{T2},7_{T1}} & & \\ & & & & & & & \theta_{8_{T2},8_{T1}} & \\ & & & & & & & & \theta_{9_{T2},9_{T1}} \end{bmatrix},$$

where $\theta_{5_{T2},5_{T1}}$ contains the covariance between the residual factors of the observed indicator X_5 ('sadness') at baseline (T1) and follow-up (T2) occasions. As only the residual factors of the same indicator are allowed to correlate over time, the matrix is a diagonal matrix (i.e. all off-diagonal elements are zero). The full $\mathbf{\Theta}$ matrix is symmetric, as $\mathbf{\Theta_{T2,T1}} == \mathbf{\Theta_{T1,T2}}$, e.g. the residual covariance $\theta_{7_{T2},7_{T1}}$ equals $\theta_{7_{T1},7_{T2}}$.

$$\boldsymbol{\mu} = \begin{bmatrix} \boldsymbol{\mu}_{\mathbf{T1}} \\ \boldsymbol{\mu}_{\mathbf{T2}} \end{bmatrix},$$

where:

$$\boldsymbol{\mu}_{\mathbf{T1}} = \begin{bmatrix} \mu_{1_{T1}} \\ \mu_{2_{T1}} \\ \mu_{3_{T1}} \\ \mu_{4_{T1}} \\ \mu_{5_{T1}} \\ \mu_{6_{T1}} \\ \mu_{7_{T1}} \\ \mu_{8_{T1}} \\ \mu_{9_{T1}} \end{bmatrix},$$

where $\mu_{1_{T1}}$ contains the mean of X_1 (i.e. the mean of the observed item scores on 'nausea') at baseline occasions (T1). In the same way $\boldsymbol{\mu}_{\mathbf{T2}}$ contains the means of the nine observed variables at follow-up occasion (T2).

$$\boldsymbol{\tau} = \begin{bmatrix} \boldsymbol{\tau}_{\mathbf{T1}} \\ \boldsymbol{\tau}_{\mathbf{T2}} \end{bmatrix},$$

where:

$$\boldsymbol{\tau}_{\mathbf{T1}} = \begin{bmatrix} \tau_{1_{T1}} \\ \tau_{2_{T1}} \\ \tau_{3_{T1}} \\ \tau_{4_{T1}} \\ \tau_{5_{T1}} \\ \tau_{6_{T1}} \\ \tau_{7_{T1}} \\ \tau_{8_{T1}} \\ \tau_{9_{T1}} \end{bmatrix},$$

where $\tau_{1_{T1}}$ contains the intercept value of X_1 ('nausea') at baseline occasion (T1). In the same way $\boldsymbol{\tau}_{\mathbf{T2}}$ contains the intercept values of the nine observed indicators at follow-up occasion (T2).

$$\boldsymbol{\kappa} = \begin{bmatrix} \boldsymbol{\kappa}_{\mathbf{T1}} \\ \boldsymbol{\kappa}_{\mathbf{T2}} \end{bmatrix},$$

where:

$$\boldsymbol{\kappa}_{\mathbf{T1}} = \begin{bmatrix} \kappa_{1_{T1}} \\ \kappa_{2_{T1}} \\ \kappa_{3_{T1}} \end{bmatrix},$$

where $\kappa_{1_{T1}}$ contains the mean of the first common factor (i.e. physical health) at baseline occasion (T1). In the same way $\boldsymbol{\kappa}_{\mathbf{T2}}$ contains the common factors means of physical-, mental-, and social health at follow-up occasion (T2).
